# Evaluation of the effects of ND: YAG laser capsulotomy on choroidal thickness and choroidal vascularity index

**DOI:** 10.1007/s10103-025-04379-x

**Published:** 2025-02-27

**Authors:** Erkut Küçük, Hüseyin Yeşilyurt, Merve Çakmak, Kürşad Razmazan Zor, Müge Çoban Karataş

**Affiliations:** Niğde Ömer Halisdemir University, Niğde, Turkey

**Keywords:** Nd-YAG laser capsulotomy, Posterior capsular opacification, Choroidal vascularity index, Choroidal thickness, Swept-source OCT

## Abstract

Posterior capsular opacification (PCO) is a common complication after cataract surgery, which is commonly treated using neodymium: yttrium–aluminum–garnet (Nd-YAG) laser capsulotomy. The purpose of this study is to investigate the impact of Nd-YAG laser capsulotomy on retinal and choroidal structures. Thirty-four eyes from 32 patients with PCO were included. Patients underwent Nd-YAG laser capsulotomy, and assessments were performed preoperatively, at 1st week, and 1st month postoperatively. OCT scans were acquired using Swept-source Optical Coherence Tomography (SS OCT). Choroidal thickness (CT) maps within the early treatment diabetic retinopathy study (ETDRS) subfields were obtained. Choroidal Vascularity Index (CVI) was calculated from OCT scans using Image J software. The preoperative, postoperative first week and first month measurements were compared. The mean age of patients was 68.7 ± 8.6 years. Visual acuity significantly improved postoperatively (*p* < 0.001), while IOP remained unchanged (*p* = 0.170). No significant change in central macular thickness (CMT) was observed across time points. CVI significantly increased postoperatively (*p* = 0.004). Choroidal thickness remained stable in most sectors, except for significant reduction in the inner inferior sector at 1 month (*p* = 0.025). Nd-YAG laser capsulotomy did not affect central macular thickness but caused localized change in choroidal thickness. Increased CVI was found which may be linked to postoperative inflammation. These findings suggest capsulotomy may affect choroidal structure and vasculature.

## Introduction

Posterior capsular opacification (PCO) is a common long-term complication of cataract surgery, leading to a decline in visual acuity for many patients [1]. Despite advancements in the techniques and material used, the incidence of PCO in adults remains between 8% and 34.3% [2]. The primary and most successful treatment for PCO is the use of a high-energy Nd: YAG laser to disrupt part of the posterior capsule, clearing the visual axis to improve vision [3]. This procedure can lead to complications, including intraocular lens (IOL) damage, IOL subluxation, post-operative increases in intraocular pressure (IOP), corneal damage, iritis, iris injury, hyphema, disruption of the anterior vitreous face. There are also complications regarding posterior segment including vitreous prolapse, retinal hemorrhage, retinal detachment, cystoid macular edema (CME), vitritis, choroidal effusion and suprachoroidal hemorrhage [4–8]. Although rare, posterior segment complications suggest that the retina and choroidal structures can be impacted by this procedure.

There are studies investigating the effect of Nd: YAG laser capsulotomy on the retina, but fewer have focused on the choroid. In the majority of these studies, choroidal thickness is utilized as a key parameter for assessing choroid. The choroidal vascularity index (CVI) is a quantitative biomarker defined as the ratio of vascular area to total choroidal area developed to detect and quantify vascular changes in the choroid. Due to its noninvasive nature and high repeatability it is investigated in various retinal and choroidal conditions [9]. While choroidal thickness can be influenced by many factors, CVI remains stable, indicating that it may serve as a more robust marker for choroidal diseases [10].

In this study, we aimed to investigate the effects of Nd: YAG laser capsulotomy on the retina and choroid, specifically evaluating its impact on choroidal health through the choroidal vascularity index.

## Materials and methods

This research was conducted at Niğde Ömer Halisdemir University Training and Research Hospital in compliance with ethical standards and the principles outlined in the Declaration of Helsinki. Approval for the study was obtained from the Clinical Research Ethics Committee of Niğde Ömer Halisdemir University (No:2024/60). All participants were thoroughly informed about the procedures and written as well as verbal consent was obtained from each participant.

The study included thirty four eyes of thirty-two patients who had undergone uncomplicated cataract surgery with single-piece intraocular lens implantation at least six months earlier, experienced reduced visual acuity due to posterior capsule opacification (PCO), and treated with Nd: YAG laser capsulotomy.

The exclusion criteria included patients with intermediate or advanced age-related macular degeneration (AMD), a history of other chorioretinal diseases, diabetic retinopathy, previous retinal treatments, glaucoma, IOP exceeding 21 mmHg, or the presence of epiretinal membranes. Patients with a spherical equivalent greater than 4 diopters, an axial length exceeding 26 mm and patients with OCT scan image quality score lower than 60 were also excluded from the study.

All participants underwent a thorough ophthalmologic examination before and after the procedure at the 1st week and 1st month which included the assessment of best-corrected visual acuity, biomicroscopic evaluation of the anterior segment, IOP measurement, fundus examination and OCT scan.

For Nd: YAG laser capsulotomy Visulas YAG (Carl Zeiss Meditec AG, Germany) was used. Patients were treated using the lowest effective energy and the minimum number of laser shots required to achieve adequate capsular opening. All capsulotomies were performed by two physicians (EK,HY). To ensure the pupil dilation, 1% tropicamide was administered before the capsulotomy. For corneal anesthesia, 0.5% proparacaine hydrochloride was applied topically. The posterior capsule was then visualized through a biomicroscope, following the placement of the Abraham capsulotomy lens. All capsulotomy openings were performed using the cross technique. After the procedure, the patient used topical fluorometholone 0.1%, four times daily for one week and topical brimonidine 0.15% twice daily for one week.

OCT scans were acquired using Triton™ DRI swept-source OCT (SS OCT) (Topcon Corporation, Tokyo, Japan). All scans were conducted at the same time each day, between 14:00 PM and 16:00 PM, to reduce the risk of fluctuations in choroidal thickness caused by diurnal variations. OCT scan image quality scores of 60 or better were included in the study.

### Analysis of OCT images

**Fig. 1:**
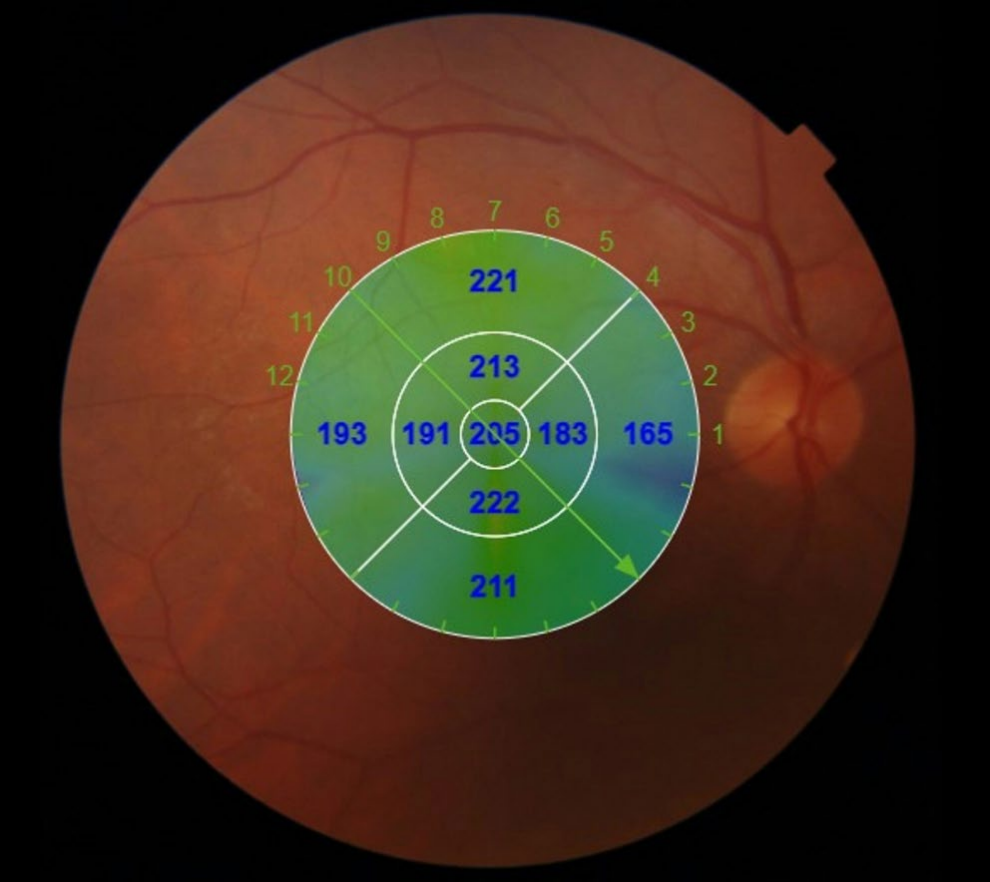
Automated choroidal thickness map of the patient generated from OCT images, utilizing the ETDRS grid to display choroidal thickness across different sectors

A radial macular scan was conducted using a 1024 × 12 scan protocol, which involves 12 radial scan lines centered on the fovea, each consisting of 1024 A-scans. Each of these lines comprised 1024 A-scans, each with a length of 6 mm. The scans were analyzed using IMAGEnet 6 software (Topcon Medical Systems, Inc). Choroidal thickness measurements were acquired using the automated software of the SS OCT device. The software generated thickness maps based on the conventional Early Treatment Diabetic Retinopathy Study (ETDRS) grid (Fig. 1). This grid comprises inner and outer rings with diameters of 1 to 3 mm and 3 to 6 mm, respectively, divided into four quadrants: superior, inferior, temporal, and nasal forming nine independent sectors. The sectors include inner and outer sectors for each of the temporal, superior, inferior, and nasal regions, along with a central sector. Choroidal segmentation was visualized using the ‘CSI’ function, which delineates the inner and outer limits of this vascular layer—Bruch membrane (BM) and choroidal scleral interface (CSI), respectively. In instances where the investigators deemed these boundaries inaccurately delineated, manual corrections were applied. Choroidal thickness (CT) values in the nine different ETDRS sectors were recorded, and the mean CT was calculated by determining the arithmetic mean of the choroidal thickness across all ETDRS grid fields.

**Fig. 2:**
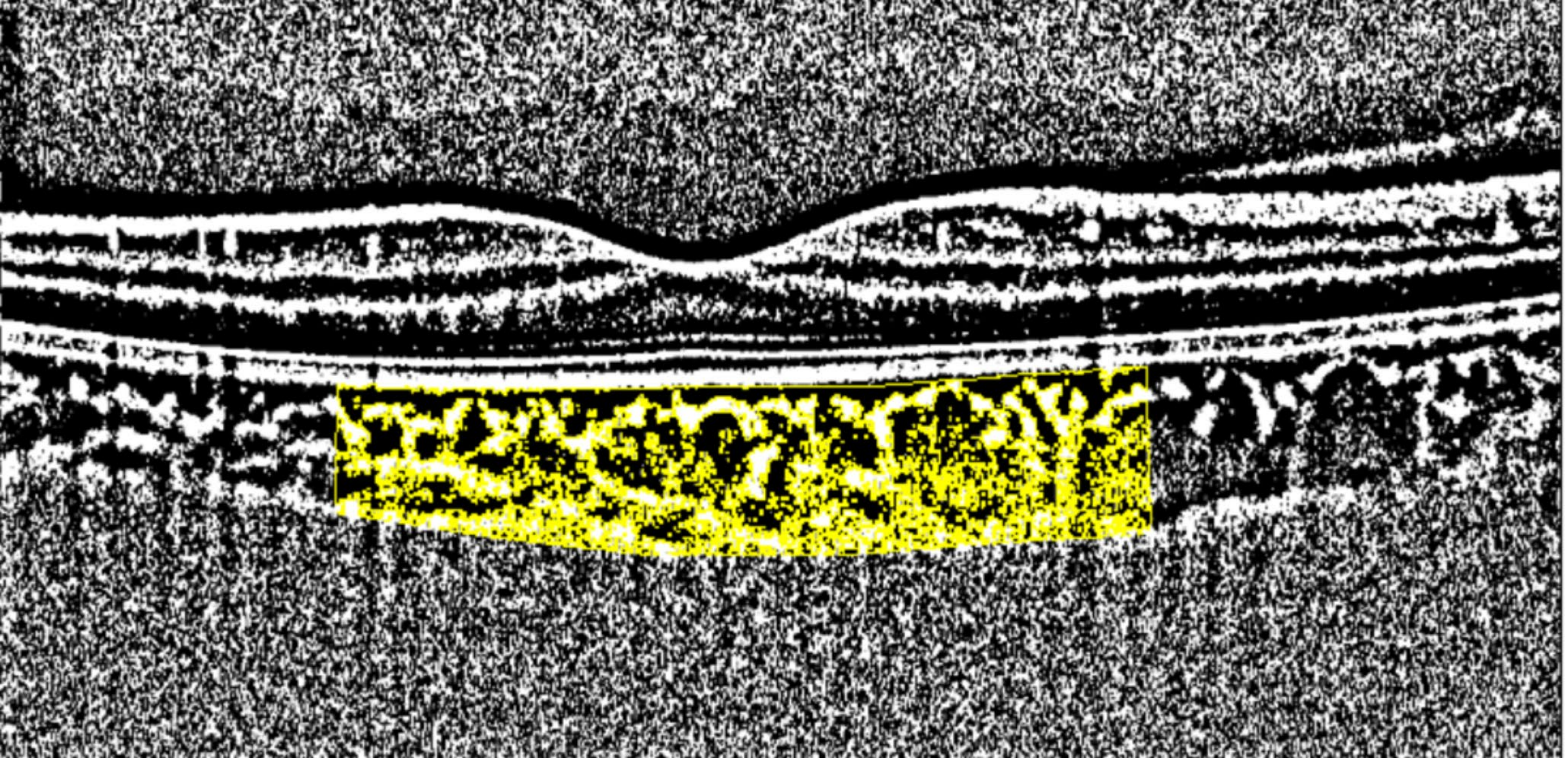
The binarized OCT image of a patient, the region of interest (ROI) was selected for analysis

CVI was measured on a foveal horizontal OCT scan with binarization using ImageJ software (https://imagej.net/Fiji/Downloads) according to the method described by Agrawal et al. [10]. Briefly, a subfoveal choroidal area, 3 mm in width and centered at the fovea, was chosen as the designated region of interest. The grey scale images were converted to binarized images using Niblack’s autolocal threshold technique. The choroidal area was delineated by marking the upper border at the RPE and the lower border at the line of light pixels at the choroid-scleral junction. This selection was made using the polygon tool and subsequently included in the region of interest manager. The image was subsequently transformed into RGB (red, green, blue) color format, enabling the utilization of the color threshold tool for the selection of dark pixels (Fig. 2). Following this, calculations were performed for both the TCA and the area occupied by dark pixels which constituted LA. To assess the choroidal vascularity status, CVI was calculated by dividing LA by TCA.

### Statistical analysis

The statistical analysis was performed utilizing SPSS version 20.0 (IBM Corporation, Armonk, NY). Descriptive statistics for quantitative data were reported as means ± standard deviations, while qualitative data were presented as percentages. The normality of the data was assessed using the Shapiro-Wilk test. To evaluate differences across the three time points, a repeated measures analysis of variance (ANOVA) was employed. Post hoc pairwise comparisons were conducted with Bonferroni correction to control for multiple testing, where appropriate. To compare image quality scores across the three time points, the Friedman test was employed. Covariance analysis was also performed to assess the effects of image quality score and time from cataract surgery to Nd: YAG laser capsulotomy on choroidal parameters. Pearson correlation analysis was performed to evaluate the relationship between the time from cataract surgeryto capulotomy and choroidal measurements, including mean choroidal thickness, central macular thickness, and CVI. A p-value of less than 0.05 was considered statistically significant.

## Results

Thirty-four eyes of 32 patients were included. The mean age was 68.7 ± 8.6 years. Seventeen (53.1%) of the participants were female and 15 (46.9%) were male. The mean axial length of the patients was 23.21 ± 1.33 mm. The average time from cataract surgery to Nd: YAG laser capsulotomy was 13.5 ± 8.1 months (range 6–32 months). The mean of total laser energy used was 25,3 ± 7,3 mJ. The visual acuity was significantly higher in the first week and first month compared to the preoperative measurements. (*p* < 0.001 for both comparisons) (Table 1). The IOP values measured preoperatively (13.6 ± 2.6 mmHg), at first week (12.8 ± 2.8 mmHg) and at the first month (14.0 ± 3.1 mmHg) were not significanly different (*p* = 0.170) (Table 1). The image quality score increased significantly after the treatment from 75.6 ± 7.8 preoperatively to 87.0 ± 5.8 in the first postoperative week and 87.5 ± 5.1 in the first postoperative month (*p* < 0.01). The central macular thickness (CMT) and Choroidal Vascularity Index (CVI) values were shown in Table 1. The CMT values showed no significant differences between three time points. None of the patients in the study developed cystoid macular edema (CME) after capsulotomy. CVI measurements differed significantly between three time points (*p* < 0.001). Post hoc tests using the Bonferroni correction revealed that the postoperative 1st week and postoperative 1st month CVI values were significantly higher than the preoperative CVI values (*p* = 0.005 and *p* < 0.001 respectively). The potential influences of image quality and the time between cataract surgery and capsulotomy on CVI were examined by covariance analysis. The results showed that while image quality had a significant interaction with the change in CVI levels (*p* = 0.013) the significant main effect of capsulotomy on CVI (*p* = 0.004) remained after adjusting for image quality, indicating that the observed changes were not due to decreased optical media transparency. The interaction between CVI measurements and time from cataract surgery to capsulotomy was not significant (*p* = 0.241), indicating that the duration between cataract surgery and Nd: YAG capsulotomy did not significantly impact CVI measurements.

Choroidal thickness (CT) values in nine different ETDRS sectors, the mean CT values and the temporal change in these parameters were presented in Table 2. There was no significant difference in mean and all ETDRS sector comparisons in three time points except inner and outer inferior sectors. In the inner and outer inferior sectors, the CT values at postoperative 1st month were significantly lower compared to preoperative values (*p* = 0.018 for both comparisons). Following covariance analysis to adjust the impact of image quality and the time from cataract surgery to Nd: YAG laser capsulotomy on changes in choroidal thickness (CT) values, the findings indicated that the inner inferior quadrant remained significantly thinner at postoperative 1st month compared to preoperative values (*p* = 0.025). There was no significant difference in mean and all other ETDRS sector comparisons across the three time points after adjustment. The Pearson correlation analysis revealed that the time interval from cataract surgery to Nd: YAG laser capsulotomy did not significantly correlate with mean choroidal thickness (*p* = 0.324, 0.310, 0.677), central macular thickness (*p* = 0.473, 0.053, 0.970) at any of the three time points (preoperative, first week postoperative, and first month postoperative). These findings suggest that the timing of Nd: YAG laser capsulotomy relative to cataract surgery did not significantly correlate with these choroidal parameters.

**Table 1:** The changes in visual acuity, intraocular pressure, central macular thickness and choroidal vascularity index after Nd: YAG laser capsulotomy

	Preoperative	Postoperative 1st week	Postoperative 1st month	*p* value
VA (logMAR)	0.47 ± 0.23	0.06 ± 0.09	0.02 ± 0.03	**< 0.001**
IOP (mmHg)	13.6 ± 2.6	12.8 ± 2.8	14.0 ± 3.1	0.170
CMT Mean ± SD (µm)	254.4 ± 24.7	248.3 ± 26.3	245.3 ± 25.9	0.185
CVI Mean ± SD	0.617 ± 0.012	0.630 ± 0.013	0.636 ± 0.011	**0.004**

VA: Visual Acuity, IOP: Intraocular pressure, CMT: Central macular thickness, CVI: choroidal vascularity index, SD: Standard Deviation, p-values indicate the statistical significance of the differences observed among the three groups

**Table 2:** The changes in the mean choroidal thickness and choroidal thickness in nine ETDRS sectors

Choroidal thickness	Preoperative Mean ± SD (µm)	Postoperative 1st week Mean ± SD (µm)	Postoperative 1st month Mean ± SD (µm)	*p* value
Central	256.5 ± 88.0	249.1 ± 87.6	247.4 ± 79.6	0.171
Inner-temporal	252.5 ± 87.0	241.3 ± 87.5	244.3 ± 82.9	0.055
Inner-superior	258.0 ± 80.9	250.9 ± 84.7	249.9 ± 76.8	0.338
Inner-nasal	238.5 ± 84.8	231.7 ± 87.6	223.9 ± 80.0	0.083
Inner-inferior	250.2 ± 93.0	239.6 ± 95.8	236.3 ± 88.1	**0.025**
Outer-temporal	232.0 ± 83.2	222.9 ± 82.3	226.0 ± 75.7	0.211
Outer-superior	244.5 ± 74.8	237.7 ± 74.5	236.4 ± 67.7	0.321
Outer-nasal	190.8 ± 69.1	184.9 ± 78.0	177.6 ± 70.1	0.063
Outer-inferior	227.2 ± 93.1	220.3 ± 92.4	215.6 ± 84.6	0.055
Mean	238.9 ± 81.7	230.9 ± 83.6	228.7 ± 76.0	0.071

SD: Standard Deviation, p-values indicate the statistical significance of the differences observed among the three groups

## Discussion

In this study we evaluated the change in central macular thickness, choroidal thickness and vascularity in patients with PCO who undergone Nd:YAG laser capsulotomy. The CMT did not change significantly after laser capsulotomy in the first week and in the first month compared to preoperative measurements. Previous studies also reported similar findings [11, 12]. Yuvacı et al. evaluated changes in central macular thickness (CMT) following laser capsulotomy over a 12-week follow-up period and found no significant alterations in CMT throughout the study duration. Yilmaz et al. conducted a 12-month follow-up study on patients after laser capsulotomy and similarly found no significant differences in CMT between preoperative and postoperative measurements.

Previous studies reported that the subfoveal choroidal thickness did not change significantly after laser capsulotomy [11–13]. However, most previous studies typically measured choroidal thickness at a single point using a horizontal foveal scan obtained via spectral domain OCT. In contrast, we utilized swept-source OCT, which offers better tissue penetration and enhanced visualization of choroidal structures [14]. This method enabled us to measure choroidal thickness using 12 radial scan lines centered on the fovea, producing a comprehensive choroidal thickness map based on the Early Treatment Diabetic Retinopathy Study (ETDRS) grid. Although choroidal thickness (CT) measurements in the central, mean, and most ETDRS grid sectors did not show significant changes after laser capsulotomy, the inner and outer inferior segments displayed reduced choroidal thickness values at the one-month follow-up compared to preoperative measurements. After adjusting for covariates including image quality and time interval between cataract surgery and capsulotomy, the inner inferior quadrant still remained significantly thinner postoperatively compared to preoperative measurements. We think that localized changes in choroidal thickness may occur following laser capsulotomy, which might not be detected through single-point measurements. A more extensive assessment covering a larger area may reveal subtle, localized alterations that could be overlooked in traditional single-point analyses. Reports on changes in choroidal thickness (CT) following various anterior segment surgical procedures have produced mixed results [15–17]. Some studies have shown an increase in CT, others a decrease, while some report no significant change. These discrepancies may be influenced by factors such as the type of procedure, patient characteristics, and the measurement method used. Different imaging techniques and protocols can yield varied outcomes in assessing CT changes post-surgery. These findings further support our observation of localized changes in choroidal thickness after laser capsulotomy suggesting that choroidal responses to laser capsulotomy may be heterogeneous and sector specific. The variability in results from other studies also aligns with the notion that localized alterations may be present, which are not always detectable through single-point measurements.

We observed that CVI values were higher in postoperative measurements compared to preoperative values. Icoz et al. also reported increased CVI after laser capsulotomy. Additionally, increased CVI has been documented in cases of anterior and intermediate uveitis, as well as after anterior segment surgeries [18–20]. It is especially noteworthy that anterior segment inflammation may cause an increased CVI without clinical findings of inflammations in the posterior segment [18]. This suggests that inflammatory processes and the release of cytokines can affect choroidal vessels, leading to vasodilation and engorgement of the choroidal vasculature [18, 20]. Our findings align with these reports, suggesting that inflammation induced by laser capsulotomy significantly affects the choroidal vasculature and contributes to an increase in CVI.

An important consideration is the effect of posterior capsular opacification (PCO) on choroidal visualization. PCO has been shown to influence various OCT-derived parameters, and imaging quality significantly improves after Nd: YAG laser capsulotomy [21]. To minimize this effect, we included only patients with a pre-capsulotomy image quality score above 60. As expected, image quality improved postoperatively in our study. To account for this factor and potential confounding effects we adjusted our analyses for image quality score. The significant main effect of capsulotomy on CVI remained even after this adjustment, suggesting that the observed changes were attributable to the laser procedure rather than improved optical media transparency. Additionally, image quality score did not have a significant impact on CT values in most ETDRS sectors, and the localized thinning observed in the inner inferior quadrant persisted after adjustments.

We also investigated the effect of the time interval between cataract surgery and Nd: YAG laser capsulotomy on the study parameters using correlation and covariance analysis but found no significant correlation. In population-based studies, the timing of capsulotomy relative to cataract surgery varies significantly [22]. The decision to perform capsulotomy is primarily based on the degree of posterior capsular opacification and its impact on visual acuity rather than the time elapsed since cataract surgery [23, 24]. This may explain the lack of a significant effect of the time from cataract surgery to Nd: YAG capsulotomy on our study parameters.

There are several limitations of the current study. The study involves a modest sample size, which may limit the generalizability of the findings to a wider population. Furthermore, while the one-month follow-up period offers valuable insights, it may not fully capture the long-term effects of Nd: YAG laser capsulotomy on retinal and choroidal health. Therefore, further research with a larger patient cohort and extended follow-up duration is necessary to explore these effects more comprehensively. A key strength of the study is the evaluation of choroidal thickness over a larger area from multiple sectors and the use of the choroidal vascularity index for more comprehensive choroidal assessment. Additionally, the use of swept-source OCT, which offers deeper tissue penetration and superior resolution, enhances imaging quality and allows for more detailed and accurate measurements of choroidal thickness and vascularity.

In conclusion, our study demonstrates that Nd: YAG laser capsulotomy has no effect on central macular thickness. While choroidal thickness did not significantly differ across most sectors, localized reductions were noted in the inferior segments, particularly in the inner inferior quadrant, highlighting the importance of more comprehensive assessments to detect subtle changes. The observed increase in CVI postoperatively indicates that inflammation induced by the procedure can significantly affect choroidal vasculature.

## Data Availability

The data that support the findings of this study are available from the corresponding author upon reasonable request.
